# RNAcentral: an international database of ncRNA sequences

**DOI:** 10.1093/nar/gku991

**Published:** 2014-10-28

**Authors:** The RNAcentral Consortium, Anton I Petrov, Simon J E Kay, Richard Gibson, Eugene Kulesha, Dan Staines, Elspeth A Bruford, Mathew W Wright, Sarah Burge, Robert D Finn, Paul J Kersey, Guy Cochrane, Alex Bateman, Elspeth A Bruford, Sam Griffiths-Jones, Jennifer Harrow, Patricia P Chan, Todd M Lowe, Christian W Zwieb, Jacek Wower, Kelly P Williams, Corey M Hudson, Robin Gutell, Michael B Clark, Marcel Dinger, Xiu Cheng Quek, Janusz M Bujnicki, Nam-Hai Chua, Jun Liu, Huan Wang, Geir Skogerbø, Yi Zhao, Runsheng Chen, Weimin Zhu, James R Cole, Benli Chai, Hsien-Da Huang, His-Yuan Huang, J Michael Cherry, Artemis Hatzigeorgiou, Kim D Pruitt

**Affiliations:** 1 European Molecular Biology Laboratory, European Bioinformatics Institute (EMBL-EBI), Wellcome Trust Genome Campus, Hinxton, Cambridge CB10 1SD, UK; 2 Faculty of Life Sciences, University of Manchester, Oxford Road, Manchester M13 9PT, UK; 3 Wellcome Trust Sanger Institute, Wellcome Trust Genome Campus, Hinxton, Cambridge CB10 1SD, UK; 4 Department of Biomolecular Engineering, University of California, Santa Cruz, CA 95064, USA; 5 Department of Biochemistry, University of Texas Health Science Center at San Antonio, 7703 Floyd Curl Drive, San Antonio, TX 78229-3900; 6 Department of Animal Sciences, 209 Animal Science Building on Mell Street, Auburn, AL 36849-5415, USA; 7 Sandia National Laboratories, Livermore, CA 94551, USA; 8 Department of Physiology, Anatomy, and Genetics, University of Oxford, Oxford OX1 3PT, UK; 9 Kinghorn Centre for Clinical Genomics, Garvan Institute of Medical Research, Darlinghurst, NSW 2010, Australia; 10 Laboratory of Bioinformatics and Protein Engineering, International Institute of Molecular and Cell Biology in Warsaw, Trojdena 4, 02-109 Warsaw, Poland, Laboratory of Bioinformatics, Institute of Molecular,Biology and Biotechnology, Faculty of Biology, Umultowska 89, 61-614 Poznan, Poland,; 11 Laboratory of Plant Molecular Biology, Rockefeller University, 1230 York Avenue, New York, NY 10065, USA; 12 Laboratory of Noncoding RNA, Institute of Biophysics, Chinese Academy of Sciences, Beijing 100101, China, Institute of Computing Technology, Chinese Academy of Sciences, Beijing 100190, China; 13 Institute of Basic Medical Sciences of Chinese Academy of Medical Sciences, Beijing, China, School of Basic Medicine Peking Union Medical College, 5 Dong Dan San Tiao, Beijing 100005, China, Taicang Institute of Life Sciences Information, Suzhou 215400, China; 14 Michigan State University, East Lansing, MI 48824-1325, USA; 15 Department of Biological Science and Technology, Institute of Bioinformatics and Systems Biology, National Chiao Tung University, HsinChu, Taiwan; 16 Department of Genetics, Stanford University, 3165 Porter Drive, Palo Alto, CA 94304, USA; 17 Department of Computer and Communications Engineering, University of Thessaly, Volos 38221; 18 Greece and National Center for Biotechnology Information (NCBI), National Library of Medicine, Bethesda, MD 20894, USA; 19 Department of Integrative Biology, University of Texas at Austin, Austin, TX 78712, USA

## Abstract

The field of non-coding RNA biology has been hampered by the lack of availability of a comprehensive, up-to-date collection of accessioned RNA sequences. Here we present the first release of RNAcentral, a database that collates and integrates information from an international consortium of established RNA sequence databases. The initial release contains over 8.1 million sequences, including representatives of all major functional classes. A web portal (http://rnacentral.org) provides free access to data, search functionality, cross-references, source code and an integrated genome browser for selected species.

## INTRODUCTION

In recent years, there has been a tremendous growth in the number of reported sequences of non-coding RNAs (ncRNAs). Large-scale genome sequencing has identified new representatives of well-known functional classes, but additionally, many new types of ncRNA have been reported, including piRNAs (1) and circRNAs (2). However, information about such sequences is often ‘locked up’ in the supplementary materials associated with publications, or may be referenced only through the chromosomal location of the encoding gene, making it cumbersome for biologists and bioinformaticians to extract the relevant data. To address this problem, specialist databases have been created for many types of ncRNAs to extract and abstract this information and to present it in a coordinated fashion on the web. Examples include miRBase (3), gtRNAdb (4), Rfam (5) and NONCODE (6). Additionally, for certain model species, there are specialist genome-centric databases that include ncRNAs within their scope; for example, the Saccharomyces Genome Database (SGD) (7) contains information about all ncRNA genes in the budding yeast *Saccharomyces cerevisiae*.

At present, some tools that researchers take for granted when analyzing protein sequences are not available for ncRNAs. For example, it has not been possible to carry out a sequence search of an individual ncRNA against all known ncRNAs due to the lack of a collection of ncRNAs. The equivalent operation has been fundamental for the advance of protein science. Identifying the full complement of ncRNAs for a particular species is also not possible, except for a few model organisms that have been intensively studied. Bringing all known ncRNA sequences into a common database would also enable the identification of sequences that are shared between resources and those that are only found uniquely in one resource. These comparisons should provide opportunities for linking between RNA information resources as well as providing quality control between different sources of ncRNA sequence.

The need for a comprehensive ncRNA sequence database was identified at a meeting of RNA researchers at Hinxton in 2010 (8), which highlighted the rapid growth in both ncRNA sequence and functional information. It was proposed that such a resource should utilize the expert community of RNA researchers through incorporation of data from the numerous ncRNA databases already in existence. To address these needs, and accelerate RNA research, we have developed RNAcentral, which aggregates information from a federation of ncRNA sequence databases. RNAcentral combines these resources to provide a comprehensive and consistent collection of accessioned ncRNA sequences. In addition, RNAcentral acts as a hub that allows users to navigate from RNAcentral back to the source of the RNA sequences. In the future, we plan to develop RNAcentral further to incorporate additional datatypes and information about RNA structure, sequence modifications, RNA–RNA and RNA–protein interactions, and function.

### RNAcentral Expert Databases

Databases that contribute sequence data to RNAcentral are known as Expert Databases. Ten such databases (3–5,9–16) have contributed to the current release (see Table 1 for details). The number of sequences contributed by each database and the level of quality assurance each offers varies: the European Nucleotide Archive (ENA), for example, contributes over 6.5 million sequences to RNAcentral, for which some have received manual attention, but others have been generated through unsupervised automated annotation processes. lncRNAdb (9) contributes just 62 sequences of long non-coding RNAs (lncRNAs), all of which are annotated with detailed information and references are provided for each. Thus, RNAcentral provides broad coverage of RNA sequence, while including rich and high quality annotation for a subset of sequences. We are currently in the process of incorporating further Expert Databases, and welcome contact from any ncRNA databases that would like to be included.

**Table 1.** Expert Databases from which sequence data are already incorporated into RNAcentral

Database name	Description	URL
ENA	European Nucleotide Archive; provides the complete set of ncRNA sequence data reported by the scientific community to the databases of the International Nucleotide Sequence Database Collaboration (INSDC; (17)) as part of conventional scientific best practice (10).	http://www.ebi.ac.uk/ena/
Rfam	Database of ncRNA families and *cis*-regulatory elements with a broad taxonomic coverage (5).	http://rfam.xfam.org/
RefSeq	A comprehensive, non-redundant, well-annotated set of reference sequences including genes and transcripts (11).	http://www.ncbi.nlm.nih.gov/refseq/
VEGA	Database of vertebrate gene annotation that provides a high-quality set of lncRNAs produced by manual annotation (12,13).	http://vega.sanger.ac.uk
gtRNAdb	Contains tRNA gene predictions on complete or nearly complete genomes from a broad range of species (4).	http://gtrnadb.ucsc.edu/
miRBase	miRBase is a database of published microRNA sequences and annotations (3).	http://mirbase.org/
RDP	Provides a quality-controlled, aligned and annotated set of small ribosomal subunit RNA sequences (14).	http://rdp.cme.msu.edu/
tmRNA Website	Provides information about the tmRNA molecule found in bacteria and some organelles (15).	http://bioinformatics.sandia.gov/tmrna/
SRPDB	Provides information about the signal recognition particle RNA molecule (16).	http://rnp.uthscsa.edu/rnp/SRPDB/SRPDB.html
lncRNAdb	Provides comprehensive information about experimentally characterized lncRNA molecules (9).	http://www.lncrnadb.org/

## DATA FLOW

### Architecture of RNAcentral

**Figure 1.**
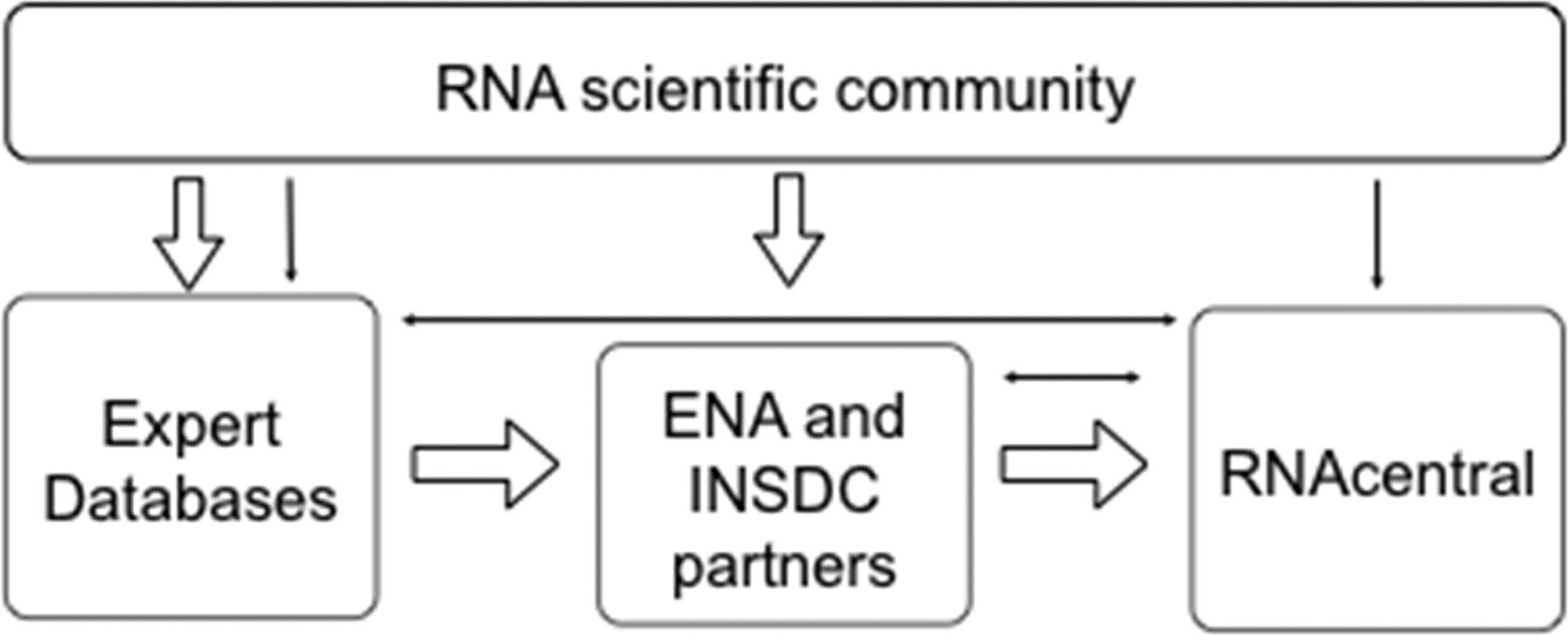
RNAcentral architecture. Block arrows indicate data flow, while solid arrows indicate web traffic and quality control.

RNAcentral has been implemented with the goal of making the resource sustainable through reuse of, and extensions to, existing bioinformatics infrastructure. In particular, core sequence data flow (including data submission/capture, validation, storage and retrieval services), cross-reference maintenance and sequence similarity search, are provided using services from the ENA (10), an established data resource that provides generic sequence archiving for the scientific community. The overall architecture and data flows into RNAcentral are illustrated in Figure 1.

Content of relevance to RNAcentral is archived directly as part of ENA in one of three ways. First, Expert Database staff report their ncRNA sequence data holdings into RNAcentral, using an ENA submission process, with assistance as appropriate from RNAcentral staff. Second, general ENA users who deposit sequence data for public dissemination provide ncRNA (and other) data through ENA submission services. Third, data reported into global partner databases of the International Nucleotide Sequence Database Collaboration (INSDC; (17)), the global nucleotide sequence database of records are mirrored to ENA through automatic processes that operate on a nightly basis. Specialist services and data presentations have been engineered under RNAcentral to support the selection and routing of ncRNA sequence data from ENA into RNAcentral-specific data flows.

### Unique RNA sequence identifiers

A major roadblock for the field of RNA biology is the lack of a set of consistent and stable accessions for RNA sequences. The goal of the current stage of the project is to catalog all known ncRNA sequences. To achieve this, RNAcentral assigns Unique RNA Sequence ids (URS) to distinct RNA sequences, no matter which species they are from. This approach parallels that of the UniProt Archive (UniParc) database (18). The benefits of this design choice are that the mapping from an identifier to an exact sequence is unique and will not change over time. In addition, the design allows a rapid look up of new sequences to check whether they already exist in RNAcentral. One downside of this design is that it creates many identifiers for sets of closely related sequences. We will address this issue in future releases, as described below.

The Unique RNA Sequence identifiers have the following format: URS + a sequentially assigned 10-digit hexadecimal number (e.g. URS00000478B7). The naming scheme can accommodate more than one trillion sequences (16^10^). Once created, the URS ids cannot be modified, deleted or re-associated with a different RNA sequence. Each URS identifier is uniquely associated with a checksum computed on the uppercase DNA version of the sequence using the MD5 algorithm described in RFC 1321 (http://www.ietf.org/rfc/rfc1321.txt). These checksum values support fast lookup of identical sequences via the RNAcentral user interfaces.

### Keeping track of cross-references

Every Unique RNA Sequence is associated with one or more cross-references (xrefs) pointing to the corresponding entries in the Expert Databases (e.g. the sequence URS00000478B7 is a human SRP RNA found in the SRPDB, Rfam, RefSeq and lncRNAdb databases). A cross-reference tracking system associates the Unique RNAcentral Sequence identifiers with the accessions used by the Expert Databases. During each RNAcentral release, cross-references can be added, kept active or deactivated (when the sequence is no longer present in the Expert Database).

### Quality control

One of the most important functions of RNAcentral is to provide quality control of the incoming data. We work closely with the Expert Databases to ensure that all data are self-consistent and meet the INSDC standards. We also examine the existing INSDC data to discover entries inappropriate for RNAcentral. For example, all ncRNA features defined using the *order* location operator were filtered out because the sequences of such entries do not represent contiguous sequences.

In addition, several ‘common sense’ rules for excluding sequences from RNAcentral have been implemented:

- Sequences that are shorter than 10 nucleotides are not included because they are not likely to represent biologically relevant ncRNAs (for a comprehensive list of ncRNAs and their sizes, the reader is referred to a recent review (19)). This cutoff is currently applied to all sequences, but in the future we may develop different cutoffs for different RNA types.
- The sequences in INSDC may include ‘N’ characters to indicate that the identity of some residues has not been established. While such sequences are allowed in RNAcentral, entries where ‘N’ residues constitute more than 10% of the sequence length are filtered out. This procedure excludes ∼0.1% of candidate sequences and about 5% of sequences with at least 1 ‘N’. As a result, in this release (version 1.0), 374 705 sequences contain ‘N’ characters, half of which have only one unknown residue.

The collection of RNA annotations in one centralized location also allows for cross-database quality control measures that were not previously possible (see also Discussion section). For example, 21 microRNA sequences deposited by miRBase are simultaneously annotated as other RNA types by different Expert Databases. These sequences have been flagged for the attention of miRBase and those Expert Databases. Similarly, a number of sequences simultaneously annotated with multiple related Rfam families were identified. This has been brought to the attention of the Rfam team, and the affected RNA families will be imported in RNAcentral once the problem is resolved.

## SUBMITTING DATA TO RNAcentral

We encourage all RNA biologists who publish the identification of novel ncRNA sequences to ensure that they are submitted into one of the INSDC databases. New ncRNA sequences submitted to INSDC are automatically imported in RNAcentral, once the data satisfy the quality control criteria described above. Reasonable assistance can be provided to Expert Databases wishing to submit annotations of existing INSDC sequences. In rare cases when the data cannot be submitted to INSDC, the data may still be imported into RNAcentral as long as the sequences can be mapped to primary INSDC accessions (e.g. contigs in a genome assembly). The contact form on the RNAcentral website can be used to get in touch with the RNAcentral team regarding data submission.

## RNAcentral WEBSITE

### Website features

The RNAcentral website is available at http://rnacentral.org and enables several ways to access data. Firstly, a text search for keywords and other metadata is provided. The results of such searches are faceted such that the results can be filtered further. For example, the user can search for all human ncRNAs, and then easily filter the results to select all rRNAs. Facets are provided for Expert Database, RNA type and species. The RNAcentral website is also equipped with a sequence search interface powered by the ENA services where the user can carry out a similarity search of a query sequence against all ncRNAs found in RNAcentral. Finally all the data can be also accessed programmatically using the REST API and the FTP archive (http://rnacentral.org/downloads).

### Genome mapping

In order to put ncRNA sequences in their genomic context, it is important to map the sequences onto their genomic locations. For example, snoRNAs that are transcribed within the introns of protein-coding genes become readily apparent when viewed in a genome browser. Knowing genomic locations also enables integration with genome browsers and other bioinformatic resources that use genome coordinates for annotating ncRNAs.

Since all reference genomes are defined using INSDC-accessioned sequences and all RNAcentral sequences are based on primary INSDC accessions, it is possible to establish a mapping between the RNAcentral entries and their genomic coordinates in reference genomes.

The Ensembl Perl API (20) is used to map the low-level INSDC accessions to their top-level genomic coordinates (such as chromosomes or contigs) for a number of key species, including human, mouse, yeast, fruit fly, worm, thale cress and others (the full list of supported species is available at the RNAcentral website). Notably, all human entries are mapped to the new human genome assembly, GRCh38, including the miRBase and VEGA Expert Database datasets. The genomic coordinates of the RNAcentral entries can be downloaded in a variety of formats from the FTP site or through the REST API.

**Figure 2.**
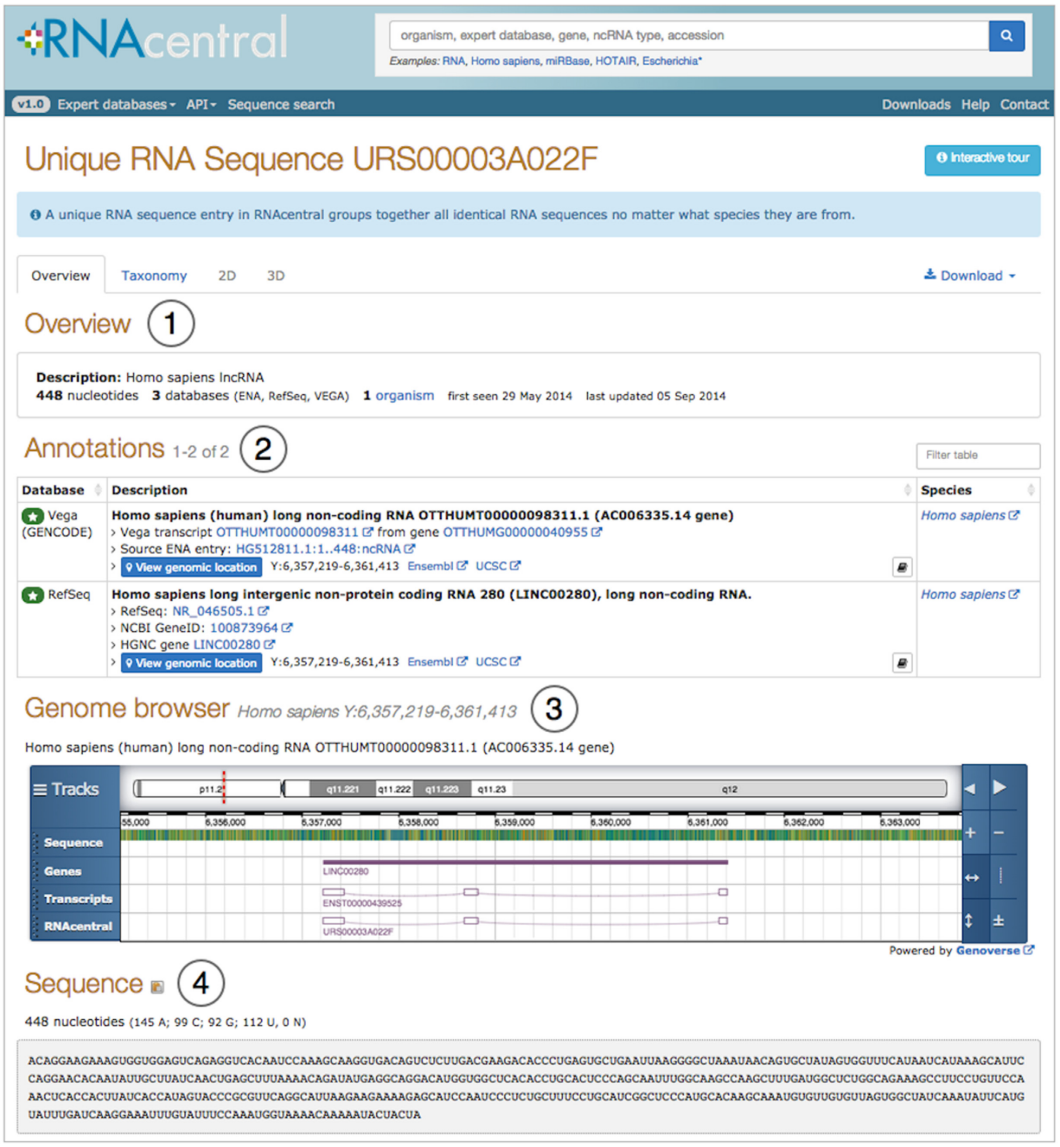
An RNAcentral entry web page for an lncRNA showing the four sections: (1) Overview and description of the RNA sequence, (2) Annotations and cross-references to Expert Databases, (3) Genome browser for mapped sequences, (4) Sequence data.

Whenever genomic mapping is available, RNAcentral sequences can be viewed in their genomic context using a light-weight genome browser (http://genoverse.org) where the users can interactively explore the genomic neighborhood without leaving the page (see Figure 2). External links are provided to the fully-featured genome browsers such as Ensembl (20) and the UCSC genome browser (21).

### Overview of the data

**Figure 3.**
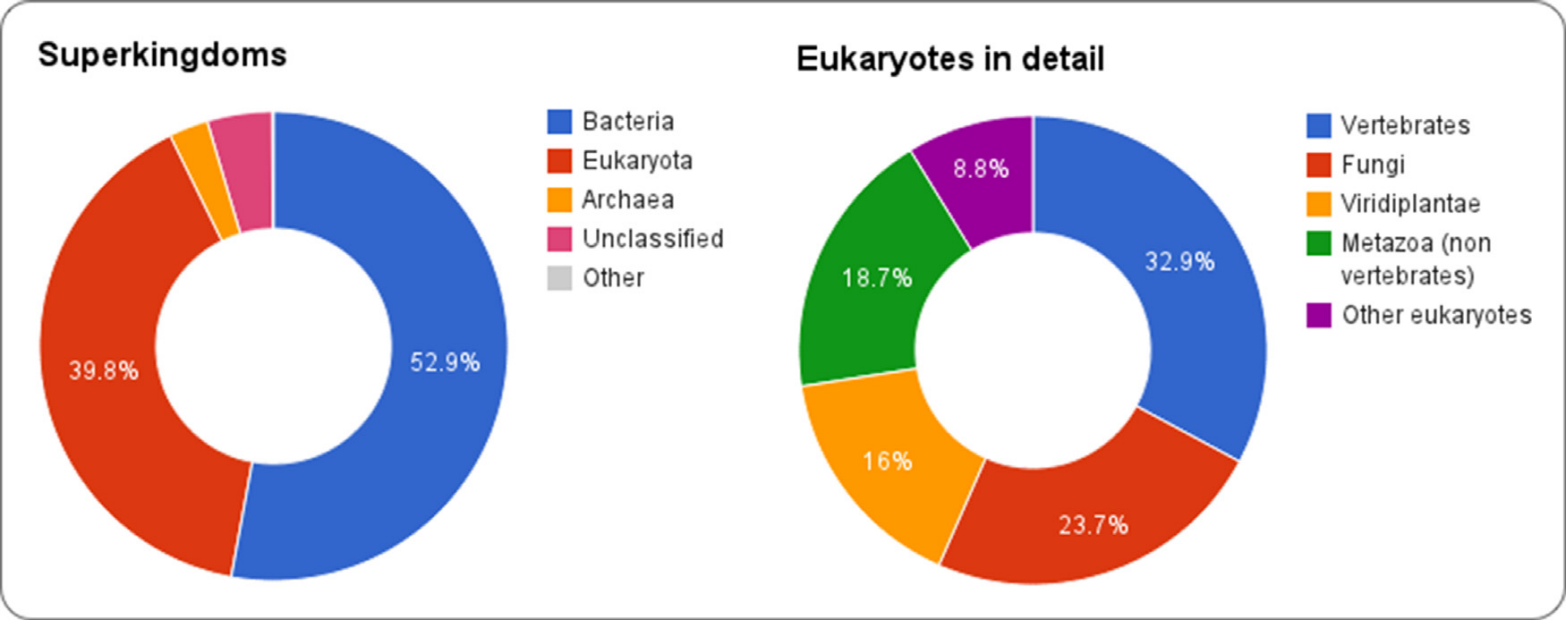
The species distribution of sequences in RNAcentral.

The current release 1.0 of RNAcentral contains over 8.1 million unique sequences. The sequences in RNAcentral are very biased toward ribosomal RNAs (70% of all sequences) that are used in environmental sampling to identify species. The class of tRNAs account for a further 10% of RNAcentral sequences. We can also look at the distribution of RNAcentral sequences across species, shown in Figure 3. Bacterial sequences account for about half of RNAcentral, while eukaryotes account for about 40% of the sequences. While there are far fewer eukaryotic genomes available, each has a larger number of RNAs. Vertebrates currently account for about one third of all eukaryotic RNAcentral sequences. In this section, we will illustrate RNAcentral data using three model organism examples.

The reference *S. cerevisiae* strain S288C (taxonomic identifier taxid:559292) contains 238 RNA sequences according to RNAcentral. SGD, the yeast model organism database, identifies 424 RNA genes leading to 191 unique sequences. In budding yeast tRNA sequences are duplicated many times. Twenty-one tRNA sequences are found twice in the genome and 13 sequences have more than 10 identical copies. There are also two complete copies of the rDNA repeats included in the reference genome. Of the 191 unique sequences in SGD, we can assign 163 (85%) as being identical to an RNAcentral sequence. Seventy-five sequences are found to be unique to RNAcentral and 28 sequences are unique to SGD. Of the 28 sequences not found in RNAcentral, all but one are encoded by the mitochondrial genome, 24 tRNAs, two rRNAs and an unclassified ncRNA sequence. The reference genome of budding yeast, strain S288C, was changed from taxid:4932 to the more specific taxid:559292 2 years ago. The source of the yeast mitochondrial RNAs has apparently not been updated to taxid:559292 and thus these mitochondrially encoded tRNAs are not associated with the proper taxid. The remaining sequence unique to SGD is SNR17A, an intron-containing gene. The intron containing form of SNR17A is present in RNAcentral, but not the intronless form. Twenty-seven of the 75 RNAcentral unique sequences contain the gene's intron sequence and thus do not represent the mature form of the RNA. As many as 47 of the RNAcentral unique sequences are likely to be due to partial matches by Rfam families creating new unique sequences. In all cases, the full-length sequence identical to SGD also exists. For example, the snoRNA snR45 from SGD is 172 nucleotides long and can be found in URS00000284F1, while Rfam provides a 171 nucleotide sequence (URS00006C1FAA) that lacks the final uracil.

A search for human ncRNAs in RNAcentral using the taxonomic identifier (taxid:9606) identifies 75 931 sequences, which exceeds the number one expects. This number includes 32 668 miscRNAs, 21 756 lncRNAs, 5139 microRNAs, 4042 rRNAs, etc. The miscRNA category contains a large number of piRNAs that have not been given the correct type by submitting authors. There appear to be twice as many microRNAs as expected (miRBase annotates ∼2500 mature microRNA sequences). The inflated number is due to many factors including multiple different sequenced versions of human DNA, which leads to multiple variants of each RNA sequence. In addition, sequences derived from the Rfam Expert database again have different 5′ or 3′ ends from the experimentally characterized ends of the microRNA meaning that completely new URS sequences are created.

The reference *Escherichia coli* strain K-12 substr. MG1655 (taxid: 511145) contains 207 ncRNA genes according to EcoCyc (22). RNAcentral identifies 367 sequences. The genome contains 7 ribosomal RNA operons (23), and in RNAcentral we find 7 full-length LSU sequences in addition to 14 shorter sequences that correspond to partial matches to the RNA. For SSU rRNA, we see only six sequences in RNAcentral. The discrepancy is explained by the fact that there are two copies of the SSU rRNA (rrnB and rrnE), which are identical in sequence and found in a single RNAcentral entry (URS00000ABFE9).

### Release schedule

The current release (1.0) follows a public beta release (1.0beta) in June 2014. In the future, the data will be updated several times a year, coinciding with major new versions of the Expert Databases. The website user interface will be updated continuously.

## DISCUSSION

RNAcentral is still at an early stage of development. The first release provides a stable accessioned set of RNA sequences, along with sequence and metadata search, bulk download, cross-references and integrated genome browsing functionality. The final goal is to develop a resource akin to UniProt for ncRNAs, with rich functional annotation and identifiers for conceptual biological entities (in addition to those assigned to sequences).

One challenge is that RNAcentral is entirely dependent on the quality of the input streams of data. For example, there are incorrect annotations of tRNA as rRNA coming from user submissions in ENA (e.g. JQ737315.1). The RNAcentral website enhances our ability to spot inconsistencies and we intend to provide automated solutions to refine the data to remove such obvious annotation errors. We will improve the provenance of sequences in RNAcentral to allow users to select slices of the data for either improved accuracy or improved coverage. In the current data scheme if an RNA sequence has even a single variant nucleotide (including an N), the two sequences will be given two different URS entries. This is far from ideal and a significant future effort will be placed on creating a new entity that groups all variants of the same ncRNA from a particular species. Further complications arise when identical RNA sequences are found at multiple genomic locations and so it will be important to also have an entity for an RNA gene that includes genomic location.

At present we are far from covering all known ncRNA sequences in RNAcentral. piRNAs, for example, are poorly represented in the database. This can be alleviated by including specialist databases such as piRNAbank in RNAcentral. We have clear future plans to incorporate several more RNAcentral Expert databases: NONCODE, CRW, plncDB, tRNAdb, sRNAmap, snoRNAdb, SILVA, GreenGenes and tmRDB. However, not every type of RNA has its own specialist database, so in the longer term we plan to contact the authors of RNA discovery papers to encourage submission of sequence data to INSDC.

A number of features for the database are planned for the coming year. These include mapping RNA sequences onto secondary and tertiary structure information. We will also incorporate the sequences of RNA from the structures in the PDB into RNAcentral, which will enable mapping of structural information to sequences in RNAcentral. We will expand mapping of RNAcentral sequences onto genomes, which provides a powerful way to understand contextual information about the RNAs.

We welcome all feedback and suggestions, which can be directed to us using the contact form on the RNAcentral website, as well as via GitHub and Twitter (the links are available at http://rnacentral.org).
